# Oral Contraceptive Use and Assessment of Breast Cancer Risk among Premenopausal Women via Molecular Characteristics: Systematic Review with Meta-Analysis

**DOI:** 10.3390/ijerph192215363

**Published:** 2022-11-21

**Authors:** Agnieszka Barańska

**Affiliations:** Department of Medical Informatics and Statistics with e-Health Lab, Medical University of Lublin, 20-094 Lublin, Poland; agnieszkabaranska@umlub.pl

**Keywords:** oral contraceptives, birth control pill, subtype breast cancer risk, ER+, ER−, HER2 positive, TNBC, case-control studies, premenopausal women, young women, breast cancer

## Abstract

Breast cancer is divided into four molecular subtypes. Each one has distinct clinical features. The aim of this study was to assess individual breast cancer subtype risk in premenopausal women taking oral contraceptives (OCs). Databases (MEDLINE; PubMed, EMBASE, and the Cochrane Library) were searched to January 2022 to identify case-control studies meeting the inclusion criteria. The influence of OCs intake on the risk of ER-positive breast cancer (ER+BC) was revealed to be non-significant with regard to reduction: OR = 0.9134, 95% CI: 0.8128 to 1.0265, *p* = 0.128. Assessment of ER-negative subtype breast cancer (ER−BC) risk indicated that OCs use significantly increased the risk: OR = 1.3079, 95% CI: 1.0003 to 1.7100, *p* = 0.050. Analysis for HER2-positive breast cancer (HER2+BC) risk showed that OCs use statistically non-significantly lowered the risk: OR = 0.8810, 95% CI: 0.5977 to 1.2984, *p* = 0.522. Meta-analysis with regard to Triplet-negative breast cancer (TNBC) risk showed non-statistically significant increased risk: OR = 1.553, 95% CI: 0.99 to 2.43, *p* = 0.055. The findings of the meta-analysis suggest that breast cancer risk in premenopausal women may vary with respect to molecular subtypes. Extensive scientific work is still necessary in order to understand the impact of OCs use on breast cancer risk in young women.

## 1. Introduction

Breast cancer is the leading type of cancer in women in most countries in the world. The incidence of breast cancer has increased dramatically in the past fifty years. Indeed, it is estimated that one in eight women will develop cancer during their lifetime. According to GLOBOCAN 2020 data, breast cancer is currently one of the most prevalently diagnosed cancers, with an estimated number of 2.3 million new cases worldwide. This figure represents 11.7% of all cancer cases globally [1,2,3,4]. Several procedures, such as preventive behaviors in general, as well as screening programs, are crucial to a possible minimization of breast cancer incidence rate and the implementation of early treatment [5].

Breast cancer remains the most common malignancy among younger patients. The incidence of breast cancer in young women is 5.6% and is rising rapidly [6]. Compared to older age groups, breast cancer in young patients is more likely to have a larger tumor size, family predisposition genes, unfavorable biological features, advanced disease at diagnosis, and, unfortunately, poor prognosis [6]. Breast cancer in young women requires special attention due to its specific morphologic and prognostic characteristics and unique aspects, including fertility preservation and psychosocial issues [7]. Diagnosing breast cancer at an earlier age may be associated with a concern that is less likely to be faced by older women [8,9,10].

Intrinsic molecular classification of subtypes of breast cancer was done according to gene expression patterns [11,12,13]. Four major intrinsic molecular subtypes have been characterized: luminal A (ER+/PR+/HER2− with low Ki67), luminal B (ER+/PR+/HER2+ with high Ki67), HER2-overexpressing (ER−/PR−/HER2+), and TNBC (ER−/PR−/HER2−) [14]. These molecular subtypes have been correlated with the clinicopathological characteristics of tumors [15,16]. The respective subtypes of breast cancer have a complex etiology that is influenced by, inter alia, endogenous endocrine and reproductive factors such as early menarche, long duration of menstruation, age (>30 years) of first pregnancy, and null parity and late menopause [17,18].

Prolonged exposure to ovarian hormones may increase the risk of breast cancer. Endogenous estrogens are involved in the development of breast tumors by stimulating proliferation, increasing the number of cell divisions, and accumulating DNA damage caused by replication, as well as generating genotoxic compounds [19,20].

Globally, around 140 million women, approximately 13% of all women between 15 and 49 years old, utilize OCs [21]. Although many investigations on the effects of OCs taking and the risk of breast cancer have been conducted, the relationship remains unresolved [22,23,24,25]. Our previous meta-analysis in the general population indicated that OCs use has diverse impacts on the risk of breast cancer when defined by molecular markers status. Summary analysis showed that each use of OCs led to a significantly increased risk of TNBC, as well as of ER−BC. There was also a significant reduction in the risk of ER+BC and a slight reduction in the risk of HER2+BC after taking OCs [26].

The aim of this study was to assess the risk of individual breast cancer subtypes in premenopausal women taking OCs.

## 2. Methods

### 2.1. Search Strategy and Selection Criteria

Based on PRISMA (Preferred Reporting Items for Systematic Reviews and Meta-Analyses) guidelines, electronic databases—MEDLINE (PubMed), EMBASE, and the Cochrane Library—were searched to identify works published to January 2022 examining the impact of oral contraception (OC) on the risk of the breast cancer molecular subtypes in premenopausal women [27,28]. The following word search terms have been used in various combinations: ‘oral contraceptives’ or ‘birth control pill’ AND ‘subtype breast cancer risk’ or ‘ER+ subtype’ or ‘ER− subtype’ or ‘HER2 positive’ or ‘TNBC’ AND ‘case-control studies’ AND ‘young women’ or ‘premenopausal women’. Relevant studies were identified using a combination of keywords in the electronic databases.

To evaluate a study’s eligibility for inclusion, titles, abstracts, and articles were reviewed independently. Two researchers independently graded the included studies with differences resolved by consensus between other investigators. A combined search was performed, looking for case-control studies (population and hospital design). The studies included in the meta-analysis provided information on the relationship between OC use and breast cancer by molecular status. The exclusion criteria were: postmenopausal women as participants; insufficient quantitative data, the results were reported as graphics; duplicate reports; case-only studies, and articles published in languages other than English.

### 2.2. Data Extraction

Data were extracted and subsequently reviewed for accuracy. From each of the included studies, the following data were extracted: (a) the first author’s last name, source of study, country, publication year, years of data collection, and the number of participants in subgroups; (b) information on the use of OCs in subgroups: ever/never, duration, age at first use, and years since last use prior to diagnosis; (c) in the original studies, different definitions and combinations of subgroup used; this meta-analysis included subtypes grouped into the four categories: ER-positive (regardless of their PR/HER2 status), ER-negative (regardless of their PR status), HER2-positive (absence of ER/PR), and Triplet-negative (absence of ER/PR/HER2).

### 2.3. Assessment of Study Quality

To evaluate the methodological quality of the included studies, we applied the Newcastle–Ottawa Scale (NOS) [27]. Each of the included studies was assessed into three major components, including selection, comparability, and exposure for case-control studies. The scale ranged from zero to nine stars, with the latter representing the highest methodological quality.

### 2.4. Statistical Analysis

Meta-analysis was performed using Statistica 13.3 software (StatSoft Poland, Kraków, Poland). The risks (odds ratios, ORs) of breast cancer in premenopausal women associated with the OC were estimated for each study. The distribution of cases and controls at risk, ORs, and 95% CI were separately identified by molecular characteristics and for OC use (ever or never). Summary risk was calculated as estimates (95% CIs) and values from single studies were combined using the random effects model (DerSimonian and Laird). Random effects incorporate an estimate of the between-study variance and tend to provide wider confidence intervals when the results of the constituent studies differ among themselves [28,29]. Heterogeneity between the studies was measured using Cochran’s Q test and I^2^. I considered the cuts off points of I^2^ suggested by Higgins et al. [28], categorized into no heterogeneity (0%), low (<25%), moderate (25–75%), and high (>75%).

The publication bias was assessed using Begg & Mazumdar’s rank correlation test and Egger’s regression test [30,31]. Funnel plot symmetry was also checked. In order to explain the possible influence of covariates, such as age at first OCs use, duration of OCs taking, and years since last OCs use prior to diagnosis, on breast cancer risk among premenopausal women, meta-regression and subgroup analyses were applied [32]. The limited number of studies available for meta-analysis prevented me from completing subgroup analyses and tests of publication bias. Subgroup analysis for variables (duration OC use, time of last use prior to diagnosis, and age at first OC use) was calculated when a minimum of the data from three studies were available.

## 3. Results

**Figure 1:**
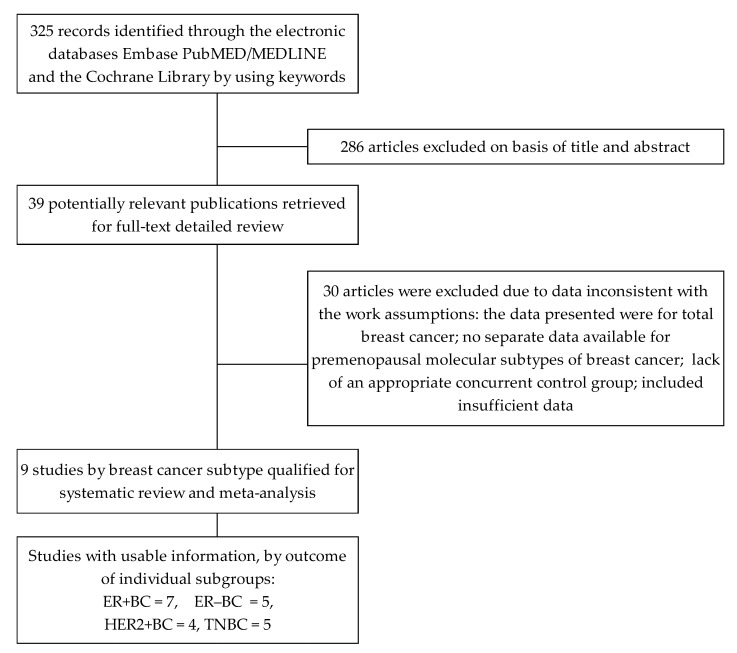
Flow diagram of literature search and research selection procedure.

Details of the search and identification process are presented in Figure 1. The search through electronic databases and relevant bibliographies identified 325 articles. Based on the titles and/or abstracts reviewed, 286 items were excluded. The remaining 39 articles with potentially relevant works were then qualified for full-text evaluation. Among these, 30 studies were, among others, excluded due to data inconsistent with the work assumptions, the data presented were for total breast cancer, and no separate data were available for pre-and postmenopausal molecular subtypes of breast cancer, or other reasons. Finally, nine articles were included in the systematic review and meta-analysis.

This analysis is based on data from nine case-control studies evaluating the effect of OCs on the risk of specific molecular subtypes of breast cancer in premenopausal women. The quality of the studies included in the meta-analysis assessed on the basis of the NOS ranged between 4 and 8, and the average score was 6.89 for the included studies.

The characteristics of the selected works are summarized in Table 1. The works were published between 1999 and 2018 and were carried out in the years 1990 to 2010. Seven studies were conducted in the United States and one each in Canada and Australia. The studies involved a total of 15,796 women, including 5973 cases of breast cancer and 9823 persons as control aged 20–50years.

The presented meta-analysis is based on data from nine studies ascertaining the effect of OCs on the risk of individual molecular subtypes of breast cancer among premenopausal women.

### 3.1. Effects of Oral Contraceptive Use on ER-Positive Subtype

**Figure 2:**
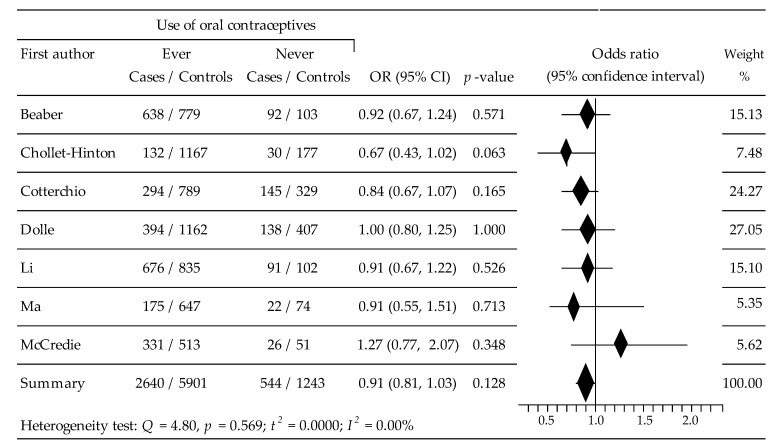
Forest plot for the association of OC intake on the risk of ER+BC.

The meta-analysis of the influence of OC intake on the risk of ER+BC was based on data from seven studies [34,35,36,37,38,39,40], which included 10,322 women (cases: 3184, control: 7144). The analysis showed that, compared to not using OCs, ever-taking OCs statistically non-significantly reduced the risk of breast cancer: OR = 0.9134, 95% CI: 0.8128 to 1.0265, *p* = 0.128, I^2^ = 0.00%. Results of Begg’s test: tau b = −0.0476, z = −0.1502, *p* = 0.881, and Egger’s test: b0 = −0.0404, 95% CI: −3.3254 to 3.2445, t = −0.0316, *p* = 0.976 indicated the lack of evidence of publication bias (Figure 2).

The assessment of the influence of OC intake on modifying factors of ER+BC risk is presented in Table 2. Data from 4 studies [35,36,37,38] investigating the duration of OC use showed an insignificant increase of ER+BC risk for use longer than 5 years, as compared with less than 5 years: OR = 0.89, 95% CI: 0.77, 1.04, *p* = 0.141, I^2^ = 00.00%. The meta-analysis of the risk of the ER+BC subtype depending on the period of discontinuation of taking of OCs before diagnosis was based on three studies [36,37,39] and was associated with a non-significant risk reduction: OR = 0.89, 95% CI: 0.64 to 1.24, *p* = 0.493, I^2^ = 52.91%. Multivariable meta-regression with covariates of the age of first use of OCs (β = 0.03, 95% CI: −0.32 to 0.38, *p* = 0.860), duration of use of OCs (β = 0.06, 95% CI: −0.16 to 0.27, *p* = 0.602), and time since last use (β = 0.25, 95% CI: −0.14 to 0.64, *p* = 0.212) showed these covariates had a non-significant impact on breast cancer risk in premenopausal women.

### 3.2. Effects of Oral Contraceptive Use on ER-Negative Subtype

**Figure 3:**
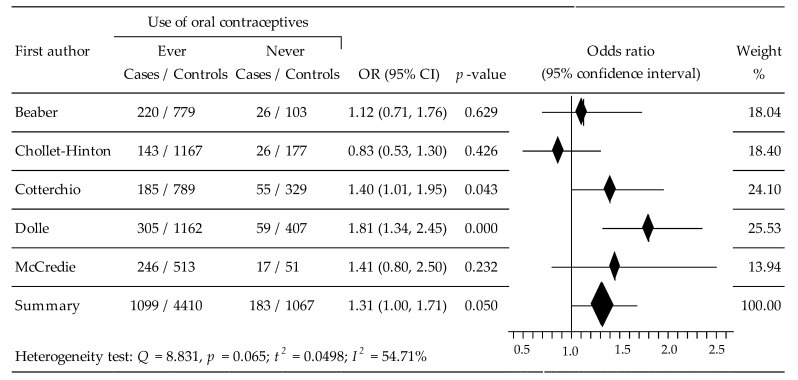
Forest plot for the association between OC use and the risk of ER−BC.

The results of the analysis regarding the association between OC use and the risk of ER−BC subtype were obtained from five studies [34,35,36,39,40], which included 6760 women (cases; 1282, control: 5477). The summary meta-analysis showed that ever-use of OCs generated significantly increased breast cancer risk, compared to non-taking OC: OR = 1.3079, 95% CI: 1.0003 to 1.7100, *p* = 0.050, with relatively moderate heterogeneity: I^2^ = 54.71% (Figure 3). No evidence of publication bias was seen after the application of Begg’s test: au b = −0.4000, z = −0.9798, *p* = 0.327, and Egger’s test: b0 = −3.6523, 95% CI; −12.1087, 4.8040, t = −1.3745, *p* = 0.263.

The influence of confounding factors on the first use of OC on the risk of ER–BC subtype could not be assessed as only one study was available for age [36], and two studies for the duration of OC use [35,36] and years since last use [36,39] (Table 2). Multivariable meta-regression duration of OC use did not confirm the impact of these covariates on this breast cancer subtype: β = −0.1832, 95% CI: −0.6131, 0.2464, *p* = 0.404. Meta-regression of years since last OCs use indicated no impact: β = 0.1061, 95% CI: −0.3481; 0.5603, *p* = 0.647.

### 3.3. Effects of Oral Contraceptive Use on HER2-Positive Subtype

**Figure 4:**
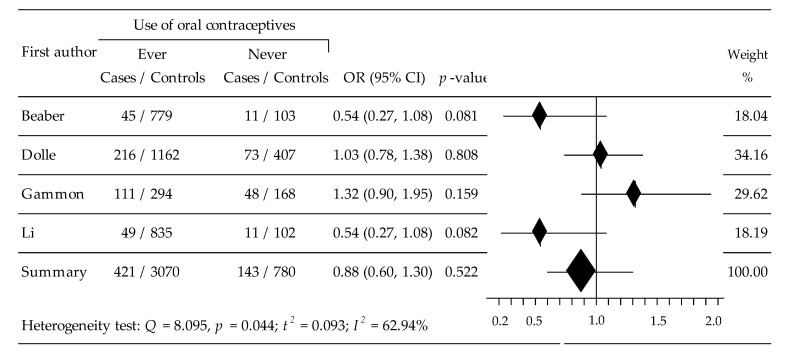
Forest plot for the relationship between OC taking and HER2+BC risk.

The relationship between OC taking and HER2+BC risk was assessed based on data from four trials [33,36,38,39] in which 4414 women participated, including 564 as cases and 3850 controls. The conducted meta-analysis showed that ever-use of OCs statistically non-significantly lowered the risk of HER2+BC: OR = 0.8810, 95% CI: 0.5977 to 1.2984, *p* = 0.522, with moderate heterogeneity: 62.94% (Figure 4). Begg’s and Egger’s tests did not show evidence of publication bias: tau b = −1.0000, z = −1.5667, *p* = 0.117, and b0 = −3.2060, 95% CI: −10,6147 to 4.2026; t = −1.8619, *p* = 0.204, respectively.

Estimation of the effect of duration of use on the risk of HER2+BC was based on data from three studies [33,36,38]. The use of OCs over 5 years was associated with a non-significant increased risk of breast cancer: OR = 1.00, 95% CI: 0.67, 1.51, *p* = 0.991, I^2^ = 58.57% (Table 2). The relation between the years from the last use of OCs before diagnosis and the risk of HER2+BC was analyzed based on data from three studies [33,36,39]. With regard to the last use of OCs >5 years, a non-significant increased risk of cancer was found: OR = 0.90, 95% CI: 0.64, 1.26, *p* = 0.537, I^2^ = 19.30% (Table 2). Multivariable meta-regression for the duration of OC use (β = 0.0186, 95% CI: −0.5998, 0.6370, *p* = 0.953), for age at first OC use (β = −0.2031, 95% CI: −0.6146, 0.2984, *p* = 0.333), and for years since last use (β = 0.2468, 95% CI: −0.1325, 0.6262, *p* = 0.202) has not confirmed the impact of these covariates on this breast cancer subtype.

### 3.4. Effects of Oral Contraceptive Use on Triplet Negative Subtype

**Figure 5:**
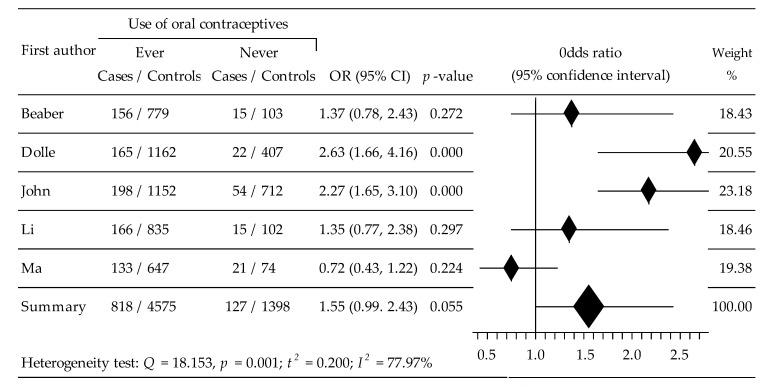
Forest plot for effects of OC use on the Triplet Negative subtype.

Changes in TNBC risk in 6918 women, including 945 cases and 5973 control, after taking OCs were assessed based upon five trials [36,37,38,39,41]. Compared with women not using OCs, the performed meta-analysis revealed non-statistically significant increased TNBC risk in women taking OCs: OR = 1.553, 95% CI: 0.99 to 2.43, *p* = 0.055, with relatively high heterogeneity, I^2^ = 77.97% (Figure 5). Begg’s test: b = 0.0000, z = 0.0000, *p* = 1.0000, and Egger’s test: b0 = −5.1703, 95% CI: −16.4055 to 6.0649, t = −1.4645, *p* = 0.239 did not show evidence of publication bias.

The analysis of the effect of the duration of OC use on the risk of TNBC was performed based on the results of three studies [36,37,38]. Meta-analysis showed a statistically insignificant increase in risk for last use <5 years: OR = 1.59, 95% CI: 0.76, 3.32, *p* = 0.218, I^2^ = 82.14% (Table 2). The influence of the time of last use prior to diagnosis on the TNBC risk was based on three studies [36,37,39]. Meta-analysis showed a statistically insignificant increase in risk for last use <5 years: OR = 1.47, 95% CI: 0.66, 3.25, *p* = 0.340, I^2^ = 81.18% (Table 2). Multivariable meta-regression for covariates duration of OC use (β = −0.279, 95% CI: −1.35, 0.818, *p* = 0.618), years since last use (β = 0.0525, 95% CI: −1.10, 1.21, *p* = 0.929), and age at the start of OC taking (β = −0.150, 95% CI: −2.062, 1.762, *p* = 0.878) did not confirm the impact of these covariates on this breast cancer subtype.

## 4. Discussion

The presented meta-analysis conducts for the first time an assessment of breast cancer risk in premenopausal women as related to molecular subtypes. The influence of OCs intake on the risk of ER-positive breast cancer among women showed that, compared to not using OCs, ever-taking OCs statistically non-significantly reduced the risk of breast cancer: OR = 0.9134, 95% CI: 0.8128 to 1.0265, *p* = 0.128. Moreover, the meta-analysis risk of ER-negative subtype breast cancer indicated that ever-use of OCs significantly increased breast cancer risk, compared to non-taking OCs: OR = 1.3079, 95% CI: 1.0003 to 1.7100, *p* = 0.050. In addition, the relationship between OC taking and HER2+BC risk showed that ever-use of OCs statistically non-significantly lowered the risk: OR = 0.8810, 95% CI: 0.5977 to 1.2984, *p* = 0.522. Finally, the performed meta-analysis of TNBC risk showed non-statistically significant increased TNBC risk in women taking OCs: OR = 1.553, 95% CI: 0.99 to 2.43, *p* = 0.055.

Our previous meta-analysis of the effect of OC use on the risk of breast cancer was conducted for the general population [26]. We included 19 studies evaluating the impact of OCs on the risk of specific molecular subtypes of breast cancer (a total of 246,152 individuals, including 31,250 cases and 214,902 as a control). The results of our meta-analysis based on the general population indicated that each use of OCs significantly increased the risk of TNBC: OR = 1.37, 95% CI; 1.13 to 1.67, *p* = 0.002, as well as ER−BC: OR = 1.20, 95% CI: 1.03 to 1.40, *p* = 0.019. The summary meta-analysis also showed a significant reduction in the risk of ER+BC: OR = 0.92, 95% CI: 0.86 to 0.99, *p* = 0.026, and a slight reduction in the risk of HER2+BC: OR = 0.95, 95% CI; 0.79 to 1.14, *p* = 0.561 after OC taking [26].

Similar results were seen in the meta-analysis of Li et al. [42] evaluating the association between OC use and TNBC risk in the general population. Results showed a significantly increased TNBC risk (OR = 1.31, 95% CI = 1.18–1.45; Z = 5.26, *p* < 0.00001). In addition, the meta-analysis of Kahlenborn et al. [43] performed for premenopausal women demonstrated that the use of OCs was associated with an increased risk of premenopausal breast cancer, in general (OR, 1.19; 95% CI, 1.09–1.29), and across various patterns of OC use. The study of Bethea et al. [44] for the overall population examined the duration of OC use in relation to molecular subtypes of breast cancer in a pooled analysis of data from the African-American Breast Cancer Epidemiology and Risk Consortium. The work included 1848 women and revealed that OC use within the previous five years was associated with increased risk of ER+BC (OR 1.46, 95% CI 1.18 to 1.81), ER- (OR 1.57, 95% CI 1.22 to 1.43), and TNBC (OR 1.78, 95% CI 1.25 to 2.53). The risk declined after cessation of use but was apparent for ER+ cancer for 15 to 19 years after cessation and ER−BC for an even longer interval after cessation. A long duration of use was also associated with an increased risk of each subtype, particularly ER−BC [44]. Results of the study of Gaudet et al. [45] suggest that both current use of contemporary OC preparations for 5 years or longer and lifetime OC durations of use of 15 years or longer confer an increased breast cancer risk among women ages 20 to 44. Our previous study evaluating the risk of breast cancer among OC users, in an overall estimate of 79 case-control studies conducted between 1960 and 2010, shows that OC use before a first full-term pregnancy significantly increased the risk of breast cancer (OR, 1.14, 1.01–1.28, *p* = 0.04), as did OC use longer than 5 years (1.09, 1.01–1.18, *p* = 0.02) [22]. A study by Bardaweel et al. conducted on 450 Jordanian women suggested that the duration of OC use was not associated with an increased risk of breast cancer (*p* > 0.05) [21]. My results also didn’t show an association between the duration of OC use and a statistically significant increased risk of breast cancer in premenopausal women.

The results of this meta-analysis should be interpreted in light of some limitations. The first may be the searching of publications submitted only in English and the fact that most of the included studies were conducted in North America. The second is that it can be difficult to define the cutoff point for the adopted age of the ‘premenopausal woman’ or ‘young woman’ categories (indeed, prior epidemiologic studies of young women’s breast cancer have used inconsistent cutoff points to classify young women–these ranging from 35 to 50 years of age). Thirdly, evidence for the associations between different OC formulations and breast cancer risk, especially by disease subtype, is limited. Fourthly, self-reporting of OC use may lead to an overestimation or underestimation of data. Fifthly, the number of cases was relatively small and previous case-control studies were based predominantly on older women. In addition, it was not reported which type of OCs they used. Moreover, there was a lack of information on confounders that could probably have had an influence on the statistical power limitations involved in examining subgroup associations.

## 5. Conclusions

The findings of the study suggest that breast cancer risk in premenopausal women may vary by molecular subtype. This conclusion prompts the suggestion that young women who use OCs should be examined more closely in population screenings of breast cancer. Unquestionably, extensive scientific work is still necessary in order to understand the impact of OC use on breast cancer risk in premenopausal women.

## Figures and Tables

**Table 1:** Characteristics of included studies.

Authors [Ref.] Publication Year	Country	Study Year	Age Range	Breast Cancer Subtype; N (n%)	NOS Score
Gammon [33] 1999	USA	1990–1992	20–40	HER2+: 159 (69.8); CRL: 462 (63.6)	4
McCredie [34] 2003	Australia	1992–1999	<40	ER+: 357 (92.7); ER−: 261 (93.5); CRL: 564 (91.0)	6
Cotterchio [35] 2003	Canada	1995–1998	<50	ER+: 439 (67.0); ER−: 240 (77.1); CRL: 1118 (70.6)	6
Dolle [36] 2009	USA	1983–1992	21–45	ER+: 532 (74.1); ER−: 364 (83.8); HER2+: 289 (74.7); TN: 187 (88.2); CRL: 1569 (74.1)	8
Ma [37] 2010	USA	1994–1998	35–44	ER+: 197 (88.8%); TN: 154 (86.4%); CRL: 721 (89.7%)	7
Li [38] 2013	USA	2004–2010	20–44	ER+: 767 (88.1); HER2+: 60 (81.7); TN: 181 (91.7); CRL: 937 (89.1)	8
Beaber [39] 2014	USA	2004–2010	20–44	ER+: 730 (87.4), ER−: 246 (89.4); HER2+: 56 (80.4); TN: 171 (91.2); CRL: 882 (88.3)	8
Chollet-Hinton [40] 2017	USA	1996–2001	<40	ER+: 162 (81.5); ER−: 169 (86.6); CRL: 1347 (86.8)	8
John [41] 2018	USA	1995–2009	<50	TN: 252 (78.6); CRL: 2223 (67.8)	7

**Table 2:** Subgroup analysis.

	Molecular Subtypes of Breast Cancer															
	ER-Positive				ER-Negative				HER2-Overexpressing				Triplet-Negative			
	OR (95% CI)	*p*	*I*^2^ (%)	Ref.	OR (95% CI)	*p*	*I*^2^ (%)	Ref.	OR (95% CI)	*p*	*I*^2^ (%)	Ref.	OR (95% CI)	*p*	*I*^2^ (%)	Ref.
	Cases/Controls				Cases/Controls				Cases/Controls				Cases/Controls			
Use of contraceptive	0.91 (0.81, 1.03)	0.128	00.00	[34,35,36,37,38,39,40]	1.31 (1.00, 1.71)	0.050	54.71	[34,35,36,39,40]	0.88 (0.60, 1.30)	0.522	62.94	[33,36,38,39]	1.55 (0.99, 2.43)	0.055	77.97	[36,37,38,39,41]
ever/never	3184/7144				1282/5477				564/3850				945/5973			
Duration of contraceptive use	0.89 (0.77, 1.04)	0.141	00.00	[35,36,37,38]	Inaccessible				1.00 (0.67, 1.51)	0.991	58.57	[33,36,38]	1.59 (0.76, 3.32)	0.218	82.14	[36,37,38]
≥5 y/<5 y	1539/3111								376/2291				464/2644			
Time last use prior to diagnosis	0.89 (0.64, 1.24)	0.493	52.91	[36,37,39]	Inaccessible				0.90 (0.64, 1.26)	0.537	19.30	[33,36,39]	1.47 (0.66, 3.25)	0.340	81.18	[36,37,39]
<5 y/≥5 y	869/2096								349/1743				368/2196			
Age at first Ocs use	Inaccessible				Inaccessible				Inaccessible				Inaccessible			

## Data Availability

Not applicable.
